# Undifferentiated Pleomorphic Sarcoma With Hyaline Globules (Thanatosomes)

**DOI:** 10.7759/cureus.15789

**Published:** 2021-06-21

**Authors:** Mitsuhiro Tachibana, Kei Tsukamoto, Mitsuru Takahashi, Yutaka Tsutsumi

**Affiliations:** 1 Department of Diagnostic Pathology, Shimada City General Medical Center, Shimada, JPN; 2 Department of Diagnostic Radiology, Shimada City General Medical Center, Shimada, JPN; 3 Department of Orthopaedic Surgery, Shizuoka Cancer Center Hospital and Research Institute, Suntou, JPN; 4 Diagnostic Pathology Clinic, Pathos Tsutsumi, Inazawa, JPN

**Keywords:** hyaline globules, thanatosomes, undifferentiated pleomorphic sarcoma

## Abstract

Hyaline globules (HGs) or thanatosomes belong to a well-defined microscopic phenomenon common to any cell type, representing eosinophilic and round-shaped intracytoplasmic inclusions as a result of altered cellular metabolism. We experienced a case of undifferentiated pleomorphic sarcoma (UPS) of the left thigh, immunoreactive diffusely for CD99 and p16^INK4a^ and focally for alpha-smooth muscle actin. HGs were multifocally clustered in the cytoplasm of the tumor cells. An ultrastructural study using a formalin-fixed, paraffin-embedded block was performed to visualize HGs in the UPS cells. Light microscopically, multifocally clustered HGs were PAS-positive with diastase-resistance and fuchsinophilic in Masson's trichrome staining. HGs were immunoreactive for cleaved caspase-3, but negative for ubiquitin. Ultrastructurally, apoptotic tumor cells contained clusters of small-sized electron-dense globules. Granular material was often deposited in the globule matrix. The formation of the HGs is supposedly related to an apoptotic process of the tumor cells. Though a nonspecific and minor microscopic finding, HGs in soft tissue sarcomas may represent a useful histologic marker of enhanced cell turnover and/or ischemic injury. This is the third report describing HGs in UPS.

## Introduction

Hyaline globules (HGs), also termed as thanatosomes, have been identified in various types of cells and tissues in the normal, subnormal, nonneoplastic and neoplastic conditions [1]. HGs represent a well-described microscopic phenomenon that may serve as a hallmark of accelerated cell turnover and apoptosis [1,2]. The term “thanatosomes” derived from the Greek words “thanatos” meaning the death and “soma” meaning the body. The presence of HGs or thanatosomes has been reported in such soft tissue malignancies as Kaposi’s sarcoma, cartilaginous neoplasms, malignant peripheral nerve sheath tumor and undifferentiated embryonal sarcoma of liver [3-6]. Undifferentiated pleomorphic sarcoma (UPS), previously known as malignant fibrous histiocytoma [7], may also accompany HGs in the cytoplasm [8,9]. We report herein a case of UPS of the left thigh, rich in HGs. Immunohistochemical and ultrastructural evaluation of HGs is described.

## Case presentation

Clinical Summary

**Figure 1:**
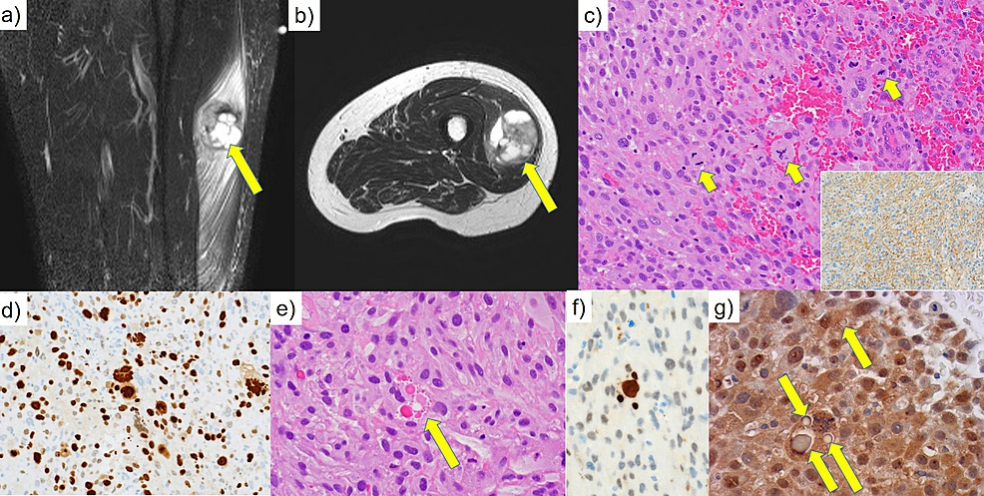
Radiological and light microscopic findings of UPS with HGs. (A,B) On magnetic resonance imaging, a T2-weighted heterogeneous intensity mass, as indicated by arrows, is seen in the skeletal muscle of the left thigh [(A) coronal section, (B) transverse section]. (C) The tumor is composed of pleomorphic spindled, stellate or epithelioid cells frequently mitotic figures (arrows) (H&E).  Bizarre nuclei are scattered. The tumor cells are immunoreactive diffusely for CD99 (MIC-2) (inset). (D)Immunostaining for Ki-67. (E) Intracytoplasmic hyaline globules (HGs) (thanatosomes) are clustered (arrow) (H&E).  Immunohistochemistry, the HGs were positive for cleaved caspase-3 (F), but negative for ubiquitin (G).

A Japanese woman nonsmoker aged in her 60’s was referred to Shimada Municipal Hospital, Shimada, Japan. She noticed an asymptomatic lump of her left thigh. Detailed radiological examinations, including magnetic resonance imaging, diagnosed the lesion as malignant soft tissue tumor (Figure 1A, 1B). A T2-weighted heterogeneous intensity mass was seen in the skeletal muscle of the left thigh. Neither lymphadenopathy nor metastasis was noted. Incisional biopsy of the lesion was performed.

Light microscopic, histochemical and immunohistochemical analysis of the biopsy material

Light microscopically, the mass was composed of pleomorphic spindled, stellate or epithelioid cells (Figure 1C), frequently accompanying foci of coagulation necrosis. Bizarre nuclei were scattered. The cytoplasm was relatively plump and eosinophilic. Mitoses, including atypical ones, were easily observed (48 in 10 high-powered fields; Figure 1C). The tumor cells were immunoreactive diffusely for CD99 (MIC-2) (Figure 1C, inset) and p16-INK4A, and moderately for a-smooth muscle actin. Negative markers included S-100 protein, desmin, heavy-caldesmon, cytokeratins, epithelial membrane antigen, p53, cyclin-dependent kinase-4 (CDK4), murine double minute-2 (MDM2), and CD34. Ki-67 labeling index was 56.9-95.8%, with the mean 62.9% (Figure 1D). Some tumor cells possessed HGs of varying sizes in the cytoplasm (Figure 1E). Their sizes ranged from 1.0 to 18.6 micrometers, with the mean value at 5.4 and median at 3.2. HGs revealed periodic acid-Schiff (PAS) reactivity with amylase resistance and fuchsinophilia in Masson's trichrome stain. The HGs were positive for cleaved caspase-3 (Figure 1F), but negative for ubiquitin (Figure 1G). The final diagnosis was UPS, accompanying HGs (thanatosomes), grade 3, according to the Federation Nationale des Centres de Lutte le Cancer.

Ultrastructural studies

A paraffin block of the biopsy specimen was processed for ultrastructural observation, as reported previously [10,11]. The specimen was routinely fixed in 0.01 M phosphate-buffered 10% formalin, pH 7.4 (Kanto Chemical, Tokyo) and embedded in paraffin (Parabett 60 GR, Muto Pure Chemicals, Tokyo). The area with tumor cells containing clustered HGs was dug out of the paraffin block as 1 mm-sized cubes. After deparaffinization overnight, and the tissue block was soaked through graded series of alcohol. The rehydrated block was re-fixed at 4C overnight in 2.5% glutaraldehyde (Yuai Kasei, Amagasaki, Japan) buffered with 0.1 M sodium cacodylate at pH 7.4, osmified for 2 hours with sodium cacodylate-buffered 1% osmium tetraoxide (Nisshin EM, Tokyo), embedded in epoxy resin (Epon 812, Okenshoji, Tokyo), and polymerized overnight in a 70C oven. Ultrathin sections were prepared using a Diatome diamond (JEOL Japan, Tokyo) at 80 nm thickness, and stained with uranyl acetate (Ieda Chemicals, Tokyo) and lead citrate (Sigma Aldrich Japan, Tokyo). Images were photographed on a JEOL JEM1400 Flash Electron microscope (JEOL Japan) equipped with an EM-14661FLASH high-sensitivity digital complementary metal-oxide-semiconductor camera.

**Figure 2:**
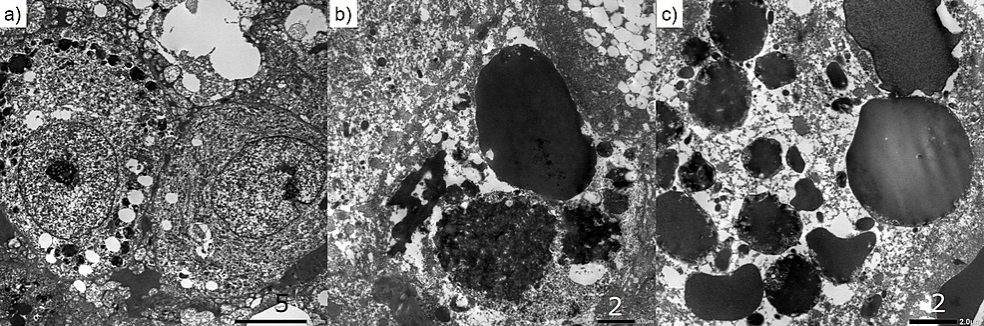
Ultrastructural findings of UPS with HGs. (a) The polygonal-shaped neoplastic cells possess round or oval nuclei with increased euchromatin and prominent nucleoli.  (b,c) HGs are formed in some tumor cells.  The globules are not homogenous, and granular material is deposited in the matrix of the globules.  The globules are not surrounded by rough endoplasmic reticulum.  Some apoptotic tumor cells contain clusters of smaller-sized electron-dense globules, ranging from 1.0 to 18.6 micrometers.  The maximal size of HGs reaches 18.6 micrometers.  [bars = 5 micrometers (a), 2 micrometers (b), 2 micrometers (c)]

Fine structural preservation was satisfactory, though not ideal. The spindled or polygonal tumor cells ultrastructurally possessed round or oval nuclei with increased euchromatin and prominent nucleoli (Figure 2A). Fine filaments were occasionally clustered in the cytoplasm. Lipid droplets were not observed. Lysosomal granules represented autophagic vacuoles. Electron-dense, round-shaped globules (HGs) were formed in the tumor cells (Figure 2B, 2C). The size of the intracytoplasmic globules ranged from 1.0 to 18.6 micrometers, with the mean value at 5.4 micrometers and median at 3.2 micrometers. The globules were not necessarily homogenous, and granular material was deposited in the matrix of the globules (Figure 2B, 2C). The globules were not surrounded by rough endoplasmic reticulum. The ultrastructural features of the globules were not typical of HGs caused by the secretory disturbance occasionally seen in epithelial cells, plasma cells or adrenomedullary cells. In combination with positive cleaved caspase-3 immunoreactivity, the HGs are supposedly related to an apoptotic process of the tumor cells, representing as genuine thanatosomes (death bodies).

Follow-up information

Neoadjuvant chemotherapy [Adriamycin+Ifosfamide (AI)] was performed for three courses in the Shizuoka Cancer Center Hospital, Nagaizumi, Shizuoka, Japan. Thereafter, the left thigh tumor was widely removed. The surgical specimen showed complete coagulation necrosis of the 65 mm-sized demarcated tumor and microscopically contained no viable tumor cells. Adjuvant chemotherapy (AI for two courses) was added. Neither recurrence nor metastasis was noted for five years.

## Discussion

The diagnosis of UPS was primarily made by anaplastic and pleomorphic histopathological features with high Ki-67 labeling index. The sarcoma cells were immunoreactive diffusely for CD99 and focally for alpha-smooth muscle actin. CD99 expression and smooth muscle differentiation in UPS have been reported [12,13]. CDK4 and MDM2, intranuclear markers of high-grade liposarcoma [14], were negative. Lipid droplets were scarcely noted ultrastructurally. In contrast, p16^INK4a^, a known immunohistochemical marker of senescence [15] and favorable prognostic marker of osteosarcoma [16] was observed, but no osteoid formation was noted in the tumor of the present case.

HGs or thanatosomes have been described in both neoplastic and nonneoplastic diseases in varied tissues and organs [1,2,17-19]. In hematoxylin and eosin-stained preparations, HGs typically appear as intracytoplasmic eosinophilic, rounded structures of varying size. HGs stain magenta with PAS reaction showing amylase-resistance, and appear fuchsinophilic in Masson’s trichrome preparation. Ultrastructurally, the HGs consisted of large osmiophilic inclusions. Papadimiriou, et al. described that the HGs ultrastructurally appeared as phagosomes/secondary lysosomes or areas of cytoplasmic condensation surrounded by rough endoplasmic reticulum whorls [1]. Double stains for apoptotic markers and plasma proteins confirmed the increased plasma membrane permeability to allow accumulation of proteins in the apoptotic cells to form HGs [1].

Cleaved caspase-3, representing an activated form of caspase-3, is an excellent and reproducible immunohistochemical marker for apoptosis [20, 21]. The biochemical pathways of apoptosis are controlled by caspases (cysteine aspartate-specific proteases), which cleave and activate a variety of intracellular proteins [22,23]. Cleaved caspase 3 functions as a kind of control tower of apoptosis: it cleaves poly (ADP-ribose) polymerase (PARP), cytokeratin 18, vimentin, actin and other intracytoplasmic proteins. Cleaved caspase 3 has been applied to detecting apoptotic neoplastic cells in paraffin sections [24-26].

In contrast, ubiquitin is ubiquitously expressed in all types of cells (hence the term ubiquitin), playing an important role in removing abnormal proteins from the cell. The ubiquitination functions as post-translational modification to control multiple steps in autophagy, a major lysosome-mediated intracellular degradation pathway [27,28]. Amorphous cytoplasmic inclusions composed of aggregated ubiquitinated intermediate filament proteins are seen in a variety of metabolic disorders such as alcoholic hepatitis and neurodegenerative diseases [29].

**Figure 3:**
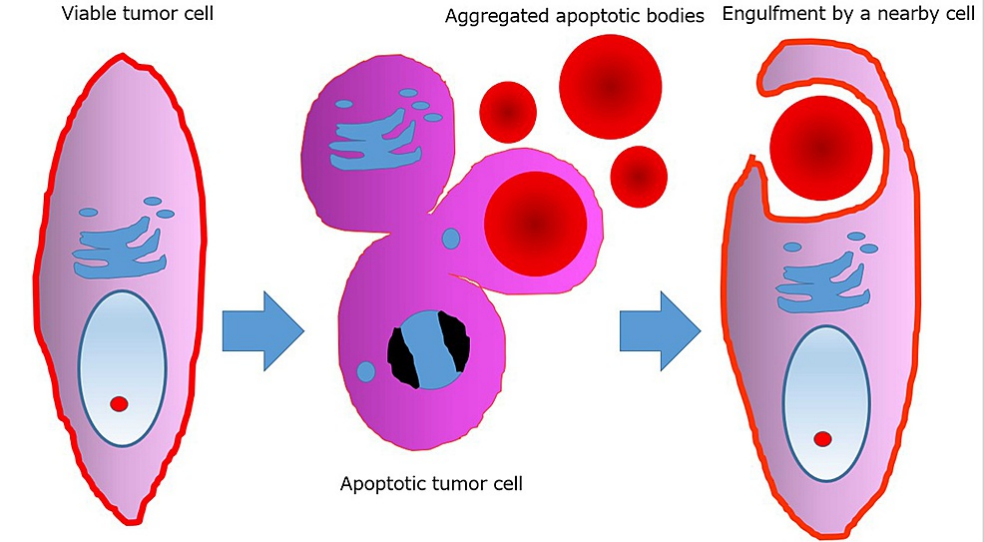
A model for the formation of HGs in the present neoplasm. An intact tumor cell responds to a variety of injurious stimuli toward apoptosis.  The apoptotic bodies are engulfed by a neighboring viable cell.

In the present analysis, the HGs in UPS were immunohistochemically positive for cleaved caspase-3, but negative for ubiquitin. Ultrastructural appearance was also compatible with the apoptosis-related process. We concluded that the HGs were formed in relation to the apoptotic process in the sarcoma cells, as illustrated in Figure 3.

There are two mechanisms for the formation of HGs. One is the above-mentioned apoptosis/ischemia-related type, and another is caused by the secretory disturbance. Apoptotic nature has been suggested in HGs seen in the benign and neoplastic gastrointestinal epithelial cells [2]. Hyaline droplets (or HGs) formed in proximal renal tubules were often related to acute tubular necrosis, and even when tubular necrosis was not evident, the proximal tubular epithelial cells containing hyaline droplets ultrastructurally showed degenerated microvilli and decreased basal interdigitations [30]. In contrast, HGs seen in plasma cells (so-called Russell bodies) [31] and HGs formed in the adrenomedullary pheochromocytes [32] typically represent the disturbance in secretory activity of the cell. del Rosario, et al. [4] reported that intracytoplasmic eosinophilic HGs in cartilaginous neoplasms were suspicious for the secretory products of probable glycoprotein nature. HG-like structures were also described in undifferentiated sarcoma cells of malignant müllerian mixed tumor of the fallopian tube. The structures displayed electron-dense membrane-bound structures that were consistent with lysosomes [33]. We recently described two cases of intraductal papillary mucinous neoplasm of the pancreas accompanying HGs of secretory disturbance type [34].

## Conclusions

To the best of our knowledge, there have been only two cases of UPS accompanying HGs, and the present study represents the first ultrastructural analysis of HGs in UPS. Further accumulation of cases is needed to clarify the incidence and clinicopathological significance of HGs in this type of high-grade sarcoma. The availability of routinely processed paraffin blocks for ultrastructural analysis should also be emphasized.
